# The Natural Flavonoid Galangin Elicits Apoptosis, Pyroptosis, and Autophagy in Glioblastoma

**DOI:** 10.3389/fonc.2019.00942

**Published:** 2019-09-27

**Authors:** Yang Kong, Zichao Feng, Anjing Chen, Qichao Qi, Mingzhi Han, Shuai Wang, Yulin Zhang, Xin Zhang, Ning Yang, Jiwei Wang, Bin Huang, Qing Zhang, Guo Xiang, Wenjie Li, Di Zhang, Jian Wang, Xingang Li

**Affiliations:** ^1^Department of Neurosurgery, Qilu Hospital of Shandong University and Institute of Brain and Brain-Inspired Science, Shandong University, Jinan, China; ^2^Shandong Key Laboratory of Brain Function Remodeling, Jinan, China; ^3^Department of Biomedicine, University of Bergen, Bergen, Norway

**Keywords:** apoptosis, autophagy, galangin, pyroptosis, glioblastoma

## Abstract

Galangin (GG), a flavonoid, elicits a potent antitumor activity in diverse cancers. Here, we evaluated the efficacy of GG in the treatment of human glioblastoma multiforme (GBM) and investigated the molecular basis for its inhibitory effects in the disease. GG inhibited viability and proliferation of GBM cells (U251, U87MG, and A172) in a dose-dependent manner (IC_50_ = 221.8, 262.5, 273.9 μM, respectively; *P* < 0.001; EdU, ~40% decrease at 150 μM, *P* < 0.001), and the number of colonies formed was significantly reduced (at 50 μM, *P* < 0.001). However, normal human astrocytes were more resistant to its cytotoxic effects (IC_50_ >450 μM). Annexin-V/PI staining was increased indicating that GG induced apoptosis in GBM cells (26.67 and 30.42%, U87MG and U251, respectively) and associated proteins including BAX and cleaved PARP-1 were increased (~3×). Cells also underwent pyroptosis as determined under phase-contrast microscopy. Knockdown of gasdermin E (GSDME), a protein involved in pyroptosis, alleviated pyroptosis induced by GG through aggravating nuclear DNA damage in GBM cells. Meanwhile, fluorescent GFP-RFP-MAP1LC3B puncta associated with autophagy increased under GG treatment, and transmission electron microscopy confirmed the formation of autophagic vesicles. Inhibition of autophagy enhanced GG-induced apoptosis and pyroptosis in GBM cells. Finally, in an orthotopic xenograft model in nude mice derived from U87MG cells, treatment with GG in combination with an inhibitor of autophagy, chloroquine, suppressed tumor growth, and enhanced survival compared to GG monotherapy (*P* < 0.05). Our results demonstrated that GG simultaneously induces apoptosis, pytoptosis, and protective autophagy in GBM cells, indicating that combination treatment of GG with autophagy inhibitors may be an effective therapeutic strategy for GBM.

## Introduction

Glioblastoma multiforme (GBM) is the most common and deadly primary malignant tumor of the central nervous system in humans. The prognosis of GBM is bleak. Median survival is 15–23 months (1), which is in part due to several biological properties rendering the tumor type particularly resistant to current therapeutic modalities. First, the blood-brain barrier (BBB) affects the absorption of drugs (2). Second, GBM cells have an intrinsic resistance to the induction of cell death (3, 4). Finally, tumors exhibit tremendous genetic heterogeneity and a complex pathogenesis so that tumors lack a single, targetable oncogenic pathway (5). The current therapeutic schedule is aggressive, including surgical resection, temozolomide (TMZ), and concurrent adjuvant radiation therapy (6), and yet, this strategy only delays tumor progression. Furthermore, it causes significant adverse reactions reducing patient quality of life. Thus, low-toxicity, effective drugs/protocols are urgently needed.

Natural flavonoids are a group of polyphenolic compounds which are ubiquitous in plants and vegetables consumed daily by humans. Flavonoids have many biological activities, which are antineoplastic, antiviral, antioxidant, and anti-inflammatory (7–10). Galangin (GG), a natural flavonoid (Supplementary Figure 1A), is an active ingredient in galangal, a spice also used in traditional Chinese medicine. GG is widespread and also found in honey and propolis. The molecule is non-toxic to humans but toxic to tumor cells making it a potential antineoplastic drug. Previous studies have investigated the antineoplastic effects of GG which appears to work through different mechanisms (8, 11, 12).

Autophagy is a cellular self-digestion process, which functions in the degradation of misfolded proteins and dysfunctional organelles (13). Substrates, such as cytoplasmic proteins or organelles, are coated by a bilayer membrane generating a vesicle called an autophagosome. Autophagosomes migrate along tracks composed of acetylated microtubules to fuse with lysosomes. The process removes the substrate through lysosomal degradation and recycles the degradation products (amino acids) to fulfill cellular metabolic needs (14). Thus, autophagy is essential for maintaining homeostasis. The process, however, has been shown to mediate resistance to anticancer therapies such as radiation, chemotherapy, and some targeted therapies (15). Many flavonoids have the effect of inducing autophagy (16, 17).

Here, we investigated the antineoplastic effect of GG and underlying molecular mechanisms in GBM cells *in vitro* and *in vivo*. Although we found that GG induces protective autophagy, we improved efficacy by combining treatment with autophagy inhibitors *in vitro* and in an orthotopic tumor model in mice. Meanwhile, we also confirmed that GG induces apoptosis and pyroptosis, two kinds of programmed cell death. Finally, we explored the interactions among autophagy, apoptosis, and pyroptosis. These results support the strategy of combination therapy using GG and autophagy inhibitors in the treatment of human GBM.

## Materials and Methods

### Experimental Animals

Male BALB/c athymic mice (4 weeks old; 14–17 g) were provided by the Nanjing Biomedical Research Institute of Nanjing University (Nanjing, China) and maintained in the animal facility for the neurosurgery laboratory of the Qilu Hospital, Shandong University under pathogen-free conditions.

### Cell Lines and Cultures

Normal human astrocytes (NHA) and human GBM cell lines, U251, U87MG, and A172, were provided by the Chinese Academy of Sciences Cell Bank (Shanghai, China). Short Tandem Repeat profiling was used to authenticate all cell lines. Mycoplasma PCR Detection Kit was used to detect mycoplasma contamination. Cells were cultured in complete medium: Dulbecco's modified Eagle's medium (DMEM; Thermo Fisher Scientific; Waltham, MA, USA) supplemented with 10% fetal bovine serum (FBS; Thermo Fisher Scientific), streptomycin (100 μg/mL) and penicillin (100 U/mL). Cells were incubated at 37°C in 5% CO_2_ in a humidified chamber.

### Cell Viability Assay

Cell viability was assessed using the Cell Counting Kit-8 assay (CCK-8; Dojindo, Kumamoto, Japan). GBM cells (4 × 10^3^ cells/well) were seeded into 96-well plates and cultured at 37°C. After 12 h the medium was replaced with 100 uL of culture medium containing different concentrations of GG (Sigma-Aldrich; MO, USA) or vehicle control (dimethyl sulfoxide, DMSO; Sigma-Aldrich, MO, USA). At 24 and 48 h after dosing, GBM cells were incubated with 10 μL of CCK-8 reagent in 100 μL of serum-free DMEM at 37°C for an hour. The absorbance at 450 nm was measured using EnSight Multimode Plate Reader (PerkinElmer; Singapore).

### Colony Formation Assay

GBM cells were seeded into 6-well plates (600 cells/well) containing 2 mL of complete medium. After cells attached, the medium was replaced with complete medium containing DMSO (control) or different concentrations of GG, and thereafter, every 3 days with fresh medium (+ treatment) over the course of the experiment. After 2 weeks, colonies were fixed with 4% paraformaldehyde, stained with 0.5% crystal violet for 15 min, and rinsed with phosphate buffer solution (PBS) three times. Colonies (> 50 cells) were counted under bright field microscopy.

### Cell Proliferation Assay

Incorporation of 5-ethynyl-2'-deoxyuridine (EdU), a thymidine analog, into proliferating cells, was detected through a catalyzed reaction between EdU and Apollo fluorescent dyes using the EdU incorporation assay (Ribobio, C103103; Guangzhou, China). Nuclei were counterstained with DAPI. EdU-positive cells in three visual fields were counted under fluorescence microscopy per hole (Leica, Dmi8; Solms, Germany).

### Protein Lysates and Immunoblotting

GBM cells were treated for 48 h and then lysed for 30 min in RIPA Lysis Buffer (Beyotime; Shanghai, China) supplemented with phenylmethanesulfonyl fluoride (PMSF, Beyotime; Shanghai, China). Cells were sonicated to enhance lysis. Lysates were centrifuged, and protein concentrations of the supernatants were determined using the BCA assay according to the manufacturer's instructions (Beyotime; Shanghai, China).

Proteins lysates (20 μg) were separated using 10–12% sodium dodecyl sulfate polyacrylamide gel electrophoresis (SDS-PAGE). The separated proteins were transferred to polyvinylidene difluoride (PVDF) membranes (0.22 μm, Millipore). PVDF membranes were blocked with 5% skim milk in Tris-buffered saline with Tween20 (TBST, 20 mmol/L Tris-HCL pH 8.0, 150 mM NaCl, 0.1% Tween-20 or with 5% BSA in TBST for phosphoproteins) for 1 h at room temperature. The membrane was incubated with primary antibodies overnight at 4°C followed by incubation with corresponding species appropriate secondary antibodies (1:2,000) for 1 h at room temperature. The following antibodies were used: AMPKα, phospho-AMPKα (Thr172; P-AMPK), mTOR, phospho-mTOR (Ser2448; p-mTOR), CDH2, CDK4, CCND1, P21, SQSTM1, BECN1, phospho-ACC (Ser79; P-ACC), and phospho-histone H2A.X (Ser139; p-H2AX; Cell Signaling Technology; Danvers, MA, USA); MAP1LC3B, ACTB, BAX, BCL-2, MMP-2, PCNA, PDK2, HMGCR, GSDMD, GSDME, Ki67, and cleaved PARP1 (Abcam; Cambridge, UK). HRP-labeled goat anti-rabbit and goat anti-mouse secondary antibodies purchased from Zhongshan Golden Bridge Bio-technology (Beijing, China). Luminous intensity was detected with the Chemiluminescence Imager (Bio-Rad ChemiDoc XRS+; Hercules, CA, USA) according to the manufacturer's protocol.

### Cell Cycle and Apoptosis Assays

GBM cells (4 × 10^5^) were seeded in 6-well plates. After incubation overnight, the culture medium was replaced with fresh complete medium with vehicle control (diluted DMSO) or GG (150 μmol/L) for 48 h. Cells were harvested through digestion with 0.05% Trypsin-EDTA (Thermo Fisher Scientific, MA, USA), incubated in cold 75% ethanol at 4°C overnight, pelleted, stained with propidium iodide for 20 min (BD Biosciences; San Jose, CA, USA), and subjected to flow cytometry for cell cycle analysis. ModFit software (Becton Dickinson; San Diego, CA, USA) was used to determine cell cycle distribution.

The fluorescein-isothiocyanate-conjugated Annexin V and PI double staining kit (BD Pharmingen; San Diego, CA, USA) was used to distinguish between early and late stage apoptosis. Briefly, GBM cells were harvested and resuspended in 1× binding buffer and stained with fluorescent dyes according to the manufacturer's protocol. Results were analyzed with Flowjo Software (Tree Star; Ashland, OR, USA).

### Quantitative Real-Time PCR (qRT-PCR)

Total RNA was prepared from treated cells using TRIzol (Thermo Fisher Scientific; MA, USA). Briefly, after centrifugation, the aqueous layer was transferred to a new eppendorf tube, and isopropanol was added to precipitate total RNA. cDNA was generated from total RNA (1–2 μg) using the ReverTra Ace qPCR RT Kit (TOYOBO; Osaka, Japan). qRT-PCR was performed with SYBR Green Master (Roche; Basel, Switzerland) on the 480II Real Time PCR Detection System (Roche; Basel, Switzerland). *ACTB* mRNA was used to normalize mRNA expression. The results are representative of at least three independent experiments. The sequences of the PCR primers used are the following: *ACTB*-F 5′-CATGTACGTTGCTATCCAGGC-3′, R 5′-CTCCTTAATGTCACGCACGAT-3′; *GSMDE*-F 5′-CCCAGGATGGACCATTAAGTGT-3′, R 5′-GGTTCCAGGACCATGAGTAGTT-3′; *ACC*-F 5′-CGCCAGCTTAAGGACAACAC-3′, R 5′-GGGATGTTCCCTCTGTTTGGA-3′; *HMGCR*-F 5′-GCAGGACCCCTTTGCTTAGA-3′, R 5′-GGCACCTCCACCAAGACCTA-3′; *PDK2*-F 5′-ATCAACCAGCACACCCTCAT-3′, R 5′-GTCACACAGGAGCTTAGCCA-3′.

### Caspase-3/7 Activity Assay

The culture medium of GG-treated cells was replaced by fresh culture containing CellEvent™ Caspase-3/7 Green Detection Reagent according to the manufacturer's protocol (Thermo Fisher Scientific; MA, USA). Cells were incubated in the dark and counterstained with Hoechest 33342 (Beyotime; Shanghai, China). The number of apoptotic cells was counted under fluorescence microscopy (Leica; Solms, Germany).

### Fluorescence Detection of Autophagic Flux

To detect autophagy, cells were infected with lentivirus expressing RFP-GFP- MAP1LC3B (Genechem; Shanghai, China) according to the manufacturer's protocol, and the number of RFP-GFP-MAP1LC3B puncta were counted in GG-treated cells under laser scanning confocal microscopy (Leica,SP8; Solms, Germany).

### Transmission Electron Microscopy

Cells were fixed with 4% glutaraldehyde and post-fixed with 1% OsO_4_ in 0.1M cacodylate buffer containing 0.1% CaCl_2_ for 2 h at 4°C. The samples were then stained with 1% Millipore-filtered uranyl acetate, dehydrated in increasing concentrations of ethanol, infiltrated, and embedded in LX-112 medium. After polymerization of the resin at 60°C for 48 h, ultrathin sections were cut with an ultracut microtome (Leica; Solms, Germany). Sections were stained with 4% uranyl acetate and lead citrate, and images were obtained using a JEM-100cxII electron microscope (Kyoto, Japan).

### Lactic Dehydrogenase (LDH) Release Assay

LDH concentration in culture medium was assessed as a measure of cell membrane integrity using the LDH Release Assay Kit according to the manufacturer's instructions (Beyotime; Shanghai, China). An increase in the LDH concentration in culture medium indicates that cell membrane integrity has been compromised.

### RNA Interference

Interfering RNA sequences (siRNA) targeting GSDME (DFNA5; GenePharma Gene; Shanghai, China) were transfected into cells with Lipofectamine 2000 reagent (ThermoFisher Scientific; MA, USA) according to the manufacturer's protocol. After 4 h, fluorescently labeled RNA was used to detect transfection efficiency. Knockdown efficiency was evaluated 48 h after transfection by qRT-PCR and immunoblotting. SiRNA sequences used are the following: 5′-GCGGTCCTATTTGATGATGAA-3′.

### Immunofluorescence Staining

Cells were fixed with 4% paraformaldehyde, permeabilized with 0.5% Triton X-100 (Beyotime, Shanghai, China) in PBS, and incubated with phospho-histone H2A.X (Ser139) antibody (1:200; Cell Signaling Technology; Danvers, MA, USA) in 5% bovine serum albumin (Sigma-Aldrich; MO, USA) in PBS overnight. Primary antibody was detected with Alexa Fluor 647-conjugated anti-rabbit IgG (Beyotime; Shanghai, China). Cells were incubated in the dark with DAPI to stain nuclei. Slides were examined under fluorescence microscopy, and images were acquired using laser scanning confocal microscopy (Leica, SP8; Solms, Germany).

### Orthotopic Xenograft Model and Bioluminescence Imaging

3 × 10^5^ cells of U87MG infected with lentivirus expressing luciferase-GFP (OBiO Technology; Shanghai, China) in ten microliters of cell suspension were stereotactically implanted into the brains (1 mm posterior to the bregma and 2 mm to the right of the midline suture at a depth of 1.5 mm) of 4-week-old athymic mice (18, 19). After 7 days, tumor size was determined, and animals were divided into the following 4 groups: control, *n* = 5; GG, *n* = 5; chloroquine (CQ; Sigma-Aldrich, C6628), *n* = 5; GG + CQ, *n* = 5). Mice were, respectively, gavaged with diluted DMSO alone (control), GG (100 mg/kg/day), CQ (25 mg/kg/day) and GG (100 mg/kg/day) + CQ (25 mg/kg/day) every day. Tumor growth was examined after implantation using bioluminescence imaging (IVIS SPECTRUM, PerkinElmer; Hopkinton, MA, USA) weekly. During the imaging procedure, the mouse was given D-Luciferin, Potassium Salt D (150 mg/kg; Yeasen Biotech Co., Ltd. Shanghai, China) under isoflurane gas anesthesia. Pictures were taken every 5 min. At the end of the experiment, tumors were dissected, and frozen in liquid nitrogen or fixed in formalin for further analysis.

### Immunohistochemistry

Tumors were removed from sacrificed mice, fixed in 4% paraformaldehyde and paraffin-embedded. Paraffin-embedded samples were sectioned (4 μm) and fixed on glass slides. Epitope retrieval of sections was performed in 10 mmol/L citric acid buffer at pH7.2 heated in a microwave. Slides were subsequently incubated with the primary antibody (rabbit anti-Ki67 1:200 dilutions) at 4°C overnight followed by HRP-conjugated secondary antibody for 1 h at room temperature. Antibodies were detected using the substrate diaminobenzidine (DAB, Beyotime; Shanghai, China), and slides were counter-stained with hematoxylin (Beyotime; Shanghai, China).

### Plotting and Statistical Analysis

Each assay was performed at least three times independently. Data analysis was performed using GraphPad Prism 6.01 software (San Diego, CA, USA). Data were reported as the mean ± SD. The statistical significance of data was evaluated using Student's *t* test between two groups and one-way analysis of variance (ANOVA) among more groups. Differences were considered to be significant at the following *P*-values: ^*^P < 0.05; ^**^P < 0.01; ^***^P < 0.001.

## Results

### GG Reduces Viability and Proliferation of GBM Cells *in vitro*

**Figure 1:**
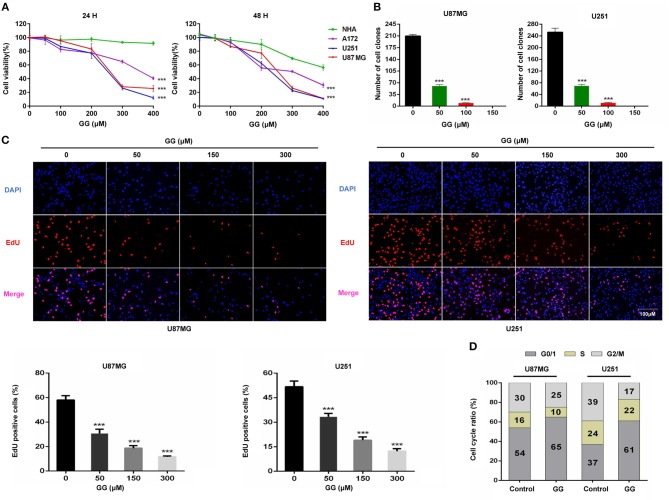
GG inhibits proliferation of GBM cells. **(A)** Graphic representation of results from CCK-8 assays to determine cell viability of U251, U87MG, A172, and NHA treated with different concentrations of GG for 24 and 48 h. Data points are the percentage (%; OD450 treated/OD450 untreated) relative to untreated cells at same time point. **(B)** Graphic representation of results from colony formation assays for U87MG and U251 under treatment with different concentrations of GG. **(C)** Fluorescence images of EdU incorporation in U87MG and U251 cells treated with GG or DMSO for 48 h. Cells were stained with Apollo 567 (red) to detect EdU and DAPI (blue) to highlight nuclei, and images were merged (magnification, 100×). Graphic representation of cell number and EdU content of U87MG and U251 treated with different concentrations of GG for 24 h. The percentage of EdU+ cells (EdU positive/DAPI positive × 100%) was determined in 4 random fields per sample. **(D)** Graphic representation of cell cycle distribution obtained using PI staining and flow cytometry. Data points are the percentage of cells in G0/1, S and G2/M in U87MG and U251 at 24 h after treatment. All data are expressed as the mean ± SD of values from experiments performed in triplicate. ***P < 0.001 compared to controls.

To begin to determine whether GG might be cytotoxic to GBM, we exposed GBM cell lines and NHA to GG *in vitro* and evaluated cell growth in several assays. Treatment with increasing concentrations of GG resulted in growth inhibition of U251, U87MG and A172 cells in a dose-dependent manner, as assessed in a cell viability assay (Figure 1A). In contrast, NHA were more resistant to treatment with increasing concentrations of GG, indicating that GG might be selective for tumor cells at certain concentrations. Increasing concentrations of GG led to decreased colony numbers in U251 and U87MG cells (Figure 1B and Supplementary Figure 1B), with no colonies appearing under treatment with 150 μM GG. These results were confirmed in EdU assays. EdU incorporation was also reduced in a dose-dependent manner in both U251 and U87MG cells treated with increasing concentrations of GG, indicating that the molecule also inhibited cell proliferation (Figure 1C). These results indicated that GG potently arrested proliferation in GBM cells in a dose-dependent manner.

### GG Induces G0/G1 Cell Cycle Arrest in GBM Cells

To determine whether GG induces cell cycle arrest in GBM cells, exponentially growing U87MG and U251 cells were treated with 150 μM GG for 24 h, and the cell cycle distribution was examined using flow cytometry. We chose to treat cells with 150 μM GG based on the results of the cell viability curves (Figure 1A), as this concentration is also non-toxic to NHA. GBM cells accumulated in G0/G1 under GG treatment compared to controls (~10–20%; Figure 1D and Supplementary Figure 1C). We next used western blotting to determine the levels of several G1/S cell cycle checkpoint proteins in GBM cells under GG treatment. Proteins associated with cell proliferation, including CCND1, CDK4, and PCNA, were reduced by ~2–3×, while a protein critical for executing G1 cell cycle arrest, cyclin-dependent kinase inhibitor p21, increased by ~2–3× (Supplementary Figures 2A,B). These results demonstrated that levels of key checkpoint proteins paralleled GG induced cell cycle arrest.

### GG Induces Apoptosis in GBM Cells

**Figure 2:**
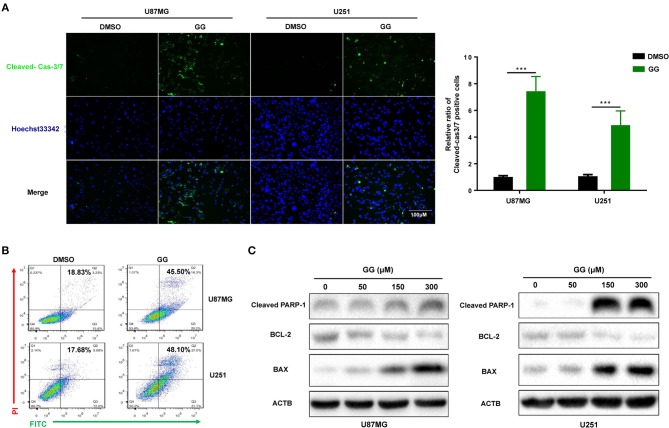
GG induces mitochondrial apoptosis and pyroptosis in GBM cells. **(A)** Fluorescence images of caspase-3 activity assay in U87MG and U251 cells treated with GG or DMSO for 48 h. Cells were stained with CellEvent™ Caspase-3/7 Green Detection Reagent (Green) to detect cleaved caspase-3 and Hoechst33342 (blue) to highlight nuclei. Images were merged (magnification, 40×). Graphic representation of the percentage of caspase-3 cleaved cells of U87MG and U251 treated with DMSO or 150 μM GG for 48 h. The percentage of caspase-3 cleaved cells (caspase-3 activated/ Hoechst33342 positive × 100%) was determined in 4 random fields per sample. **(B)** Flow cytometric analysis of ANXA5-FITC and PI staining for the determination of apoptosis in U87MG and U251 cells after treatment of DMSO or 150 μM GG for 48 h. **(C)** Western blotting analysis of lysates (20 μg) prepared from U87MG and U251 cells treated with DMSO or GG at the indicated concentrations for 48 h. Membranes were incubated with antibodies against cleaved-PARP1, Bcl-2, BAX, and ACTB (protein loading control). All data are expressed as the mean ± SD of values from experiments performed in triplicate. ***P < 0.001 compared to controls.

We next investigated whether GG induced apoptosis in GBM cells. GBM cells were treated with 150 μM GG for 48 h and first examined using an live cell apoptosis assay to detect cleaved caspase-3/7. The number of cells positive for activated caspase-3/7 increased significantly after treatment with GG compared to controls (15–20%; Figure 2A). The results were corroborated through analysis of ANXA5-FITC and PI staining of treated cells using flow cytometry. Apoptosis was significantly increased in tumor cells treated with GG relative to controls (26.67 and 30.42%, U87MG and U251, respectively; Figure 2B).

We also examined levels of apoptosis-related proteins, including Bcl-2, Bax and cleaved-PARP1, in GG-treated GBM cells by western blotting. Bcl-2, an inhibitor of apoptosis, was down-regulated in cells ~4×, while Bax and cleaved-PARP1, mediators of apoptosis, were increased ~2.5× or 30× (in a dose-dependent manner) (Figure 2C and Supplementary Figure 2C). These results indicated that apoptosis in part mediated the reduced viability of GBM cells exposed to GG.

### GG Induces Pyroptosis in GBM Cells

**Figure 3:**
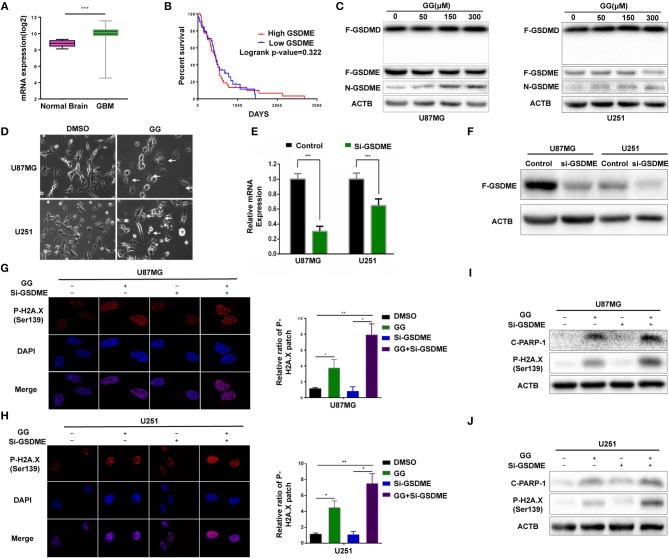
GG induces GSDME-mediated pyroptosis. **(A)** Graphic representation of the mRNA expression of *GSDME* in GBM in the TCGA database. **(B)** Kaplan–Meier survival curves for patients with GBM ^*GSDME high*^ and GBM ^*GSDME low*^ from the TCGA database. **(C)** Western blotting analysis of lysates (20 μg) prepared from U87MG and U251 cells treated with DMSO or GG at the indicated concentrations for 48 h. Membranes were incubated with antibodies against GSDMD, GSDME, and ACTB (protein loading control). **(D)** Images of U87MG and U251 cells after treatment of DMSO or 150 μM GG for 48 h under phase-contrast microscopy (magnification, 200×). Graphic representation of results from **(E)** qRT-PCR and **(F)** western blotting analysis validates the efficiency of si-GSDME. Immunofluorescence staining of p-H2A.X (Ser139) after corresponding treatment (600X) in U87MG **(G)** and U251 **(H)**. Western blotting analysis performed to detect levels of cleaved-PARP1, p-H2A.X (Ser139) and ACTB after knock-down of GSDME in U87MG **(I)** and U251 **(J)**. All data are expressed as the mean ± SD of values from experiments performed in triplicate. *P < 0.05, **P < 0.01, and ***P < 0.001 compared to controls.

Pyroptosis is a form of cell death that is critical in pathogen infection. It can be induced by canonical caspase-1 inflammasomes or through activation of caspase-4,−5, and −11 by cytosolic lipopolysaccharide (20–22). This process is mainly mediated by the gasdermin family sharing a pore-forming domain (23). Chemotherapy drugs have been reported to induce pyroptosis through caspase-3 cleavage of GSDME in primary human cells (24). To determine whether pyroptosis contributes to reduced cell viability in GBM cells, we first examined expression of *GSDME* in human glioma using the genomic data in TCGA and Rembrandt databases. GBMs expressed higher levels of *GSDME* relative to normal brain (Figure 3A). However, no significant increase in *GSDME* was associated with glioma grade in the Rembrandt database (Supplementary Figure 3A). To determine whether *GSDME* expression was associated with survival, Kaplan–Meier survival curves were generated based on the median value of *GSDME* expression in GBM in the TCGA database (http://cancergenome.nih.gov) (25). Although *GSDME* expression was significantly higher than in normal samples, overall survival (OS) was not significantly different between GBM ^high *GSDME*^ and GBM ^low *GSDME*^ (*P* = 0.322; Figure 3B). However, based on the Rembrandt database (http://www.betastasis.com/glioma/rembrandt/) (26), the survival time of GBM patients with higher expression of GSDME was significantly shorter than that of patients with lower expression (*P* = 0.022; Supplementary Figure 3B).

We next investigated the possible involvement of pyroptosis at the molecular level in GG-treated cells by western blotting. N-terminal fragment of GSDME rather than GSDMD was significantly increased in GG-treated U87MG and U251 in dose-dependent manner relative to controls (Figure 3C and Supplementary Figure 3C). Morphological features were also consistent with pyroptosis. Phase-contrast images revealed that characteristic large bubbles in the plasma membrane formed in dying cells, and whole cells displayed swelling typical of the process (Figure 3D). Finally, the release of LDH was also significantly elevated in both U87MG and U251, indicating that GG treatment interrupted the integrity of the cell membrane in GBM cells (Supplementary Figure 3D). In conclusion, both pyroptosis and apoptosis contributed to GG-induced cell death in GBM cells *in vitro*.

### Inhibition of Pyroptosis Aggravates Nuclear DNA Damage in GBM Cells

To confirm that pyroptosis in GG-treated cells was mediated by GSDME, we knocked down GSDME in U87MG and U251 using siRNA. qRT-PCR and westernbloting analyses demonstrated that siRNA efficiently knocked down GSDME at the mRNA and protein levels in both U87MG and U251 (Figures 3E,F and Supplementary Figure 3E). Growth curves generated from cell viability assays revealed no significant difference between si-GSDME and control groups (Supplementary Figure 3F). LDH release was also decreased in cells with GSDME knockdown relative to controls (Supplementary Figure 3D). As pyroptosis and apoptosis are two processes engaging programmed cell death, we investigated whether loss of GSDME affected levels of proteins typically associated with apoptosis. In cells transfected with GSDME siRNAs, the treatment of GG markedly increased the patch of p-H2AX—a marker of nuclear damage (Figures 3G,H). The results of immunoblotting further confirmed this phenomenon. Co-treatment of RNAi and GG led to increases in nuclear DNA damage-related proteins, including cleaved-PARP1 and p-H2AX (~3–5×; Figures 3I,J and Supplementary Figures 3G,H). Taken together, our results demonstrated that inhibition of pyroptosis aggravated nuclear DNA damage in GBM cells *in vitro*, indicating a possible influence of pyrostosis to the extent of apoptosis when treating glioblastoma cells with GG.

### GG Induces Autophagy in GBM Cells *in vitro*

**Figure 4:**
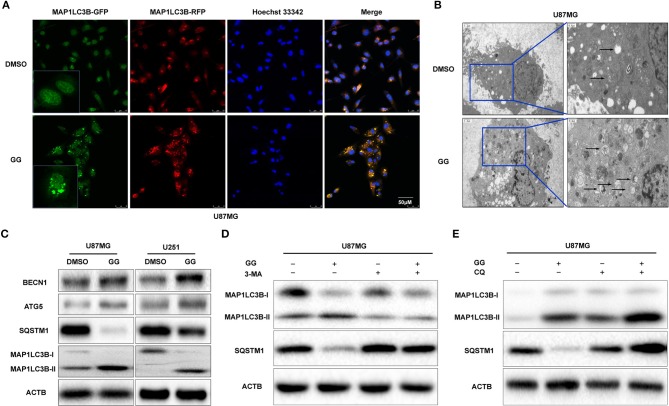
GG induces autophagy in GBM cells. **(A)** Fluorescence images of GFP-RFP-MAP1LC3B stably expressed in U87MG treated with 150 μM GG or DMSO for 48 h. The puncta visible due to GFP (green) and RFP (red) indicate formation of autophagosomes. The nuclei are stained blue with Hoechst33342. **(B)** Images from transmission electron microscopy of U87MG treated with 150 μM GG or DMSO for 48 h. The arrows highlight the autophagosomes. Scale bars: left figure 1.2 μm, right figure 0.4 μm. **(C)** Western blotting analysis performed on lysates (20 μg) to detect levels of ATG5, BCLN1, SQTM1, MAP1LC3B, and ACTB in U87MG and U251 cells after treatment of DMSO or 150 μM GG. U87MG pretreated with **(D)** 3-MA (5 mM) or **(E)** CQ (10 μM) for 20 min, followed by exposure to 150 μM GG or DMSO for another 48 h. Western blotting analysis performed to detect levels of MAP1LC3B and ACTB in U87MG. All data are expressed as the mean ± SD of values from experiments performed in triplicate.

Previous studies have suggested that GG exerts its anticancer effect by inducing autophagy (11, 27). We therefore investigated the relationship between GG and autophagy in human glioma cell lines U251 and U87MG *in vitro*. We generated U87MG cells with stable expression of GFP-RFP-MAP1LC3B. Under GG treatment, the number of MAP1LC3B fluorescent puncta increased in U87MG cells (Figure 4A). Transmission electron microscopy (TEM) is the gold standard for detecting autophagosomes, which are characterized by their double-membrane structure and contents. TEM revealed that the number of autophagosomes was increased after GG treatment (Figure 4B). Finally, the expression of MAP1LC3B-II and SQSTM1 was measured by western blotting. Alterations in the levels of these proteins, increased MAP1LC3B-II with simultaneous decreased SQSTM1, were consistent with enhanced and efficient autophagic flux (Figure 4C and Supplementary Figure 4C).

Autophagy inhibitors were used to further probe the mechanism of GG-induced autophagy. We co-treated U87MG and U251 cells with GG and 3-methyladenine (3-MA; Selleck,TX, USA) or CQ, which block early and late phases of autophagy, respectively, and examined protein levels by western blotting. Co-incubation of cells with GG and 3-MA (5 mM) for 48 h led to decreased MAP1LC3B-II. In contrast, combined treatment with GG and CQ (10 μM) led to increased expression of both SQSTM1 and MAP1LC3B-II, compared to GG treatment alone (Figures 4D,E and Supplementary Figures 4A,B,D–G). These results thus indicated that GG induced autophagy through molecules classically associated with the process.

### GG Induces Autophagy Through Activation of the AMPK/mTOR Pathway in GBM Cells

**Figure 5:**
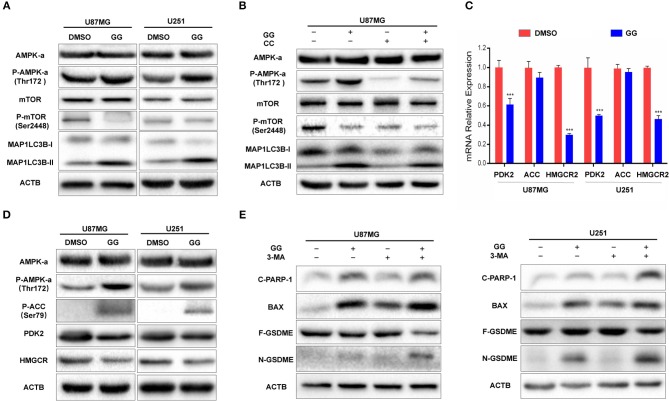
GG induces protective autophagy through activation of the AMPK/mTOR pathway. **(A)** Western blotting analysis performed on lysates (20 μg) for AMPKα, P-AMPKα (Thr172), mTOR, P-mTOR (Ser2448), MAP1LC3B and ACTB in U87MG and U251 cells treated with DMSO or 150 μM GG for 48 h. **(B)** Western blotting analysis performed on lysates (20 μg) for AMPKα, P-AMPKα (Thr172), mTOR, P-mTOR (Ser2448), MAP1LC3B and ACTB after co-incubation of U87MG cells with GG and AMPK inhibitor Compound C. **(C)** Graphic representation of results of downstream molecules of the AMPK pathway from qRT-PCR for after exposure to 150 μM GG or DMSO for 48 h. *P < 0.05, **P < 0.01, and ***P < 0.001 compared to controls. **(D)** Western blotting analysis of lysates (20 μg) prepared from U87MG and U251 cells treated with DMSO or 150 μM GG for 48 h. Membranes were incubated with antibodies against AMPKα, P-AMPKα (Thr172), P-ACC (Ser79), PDK, HMGCR and ACTB. *P < 0.05, **P < 0.01, and ***P < 0.001 compared to controls. Western blotting analysis performed to detect levels of cleaved-PARP1, BAX, GSDMD, GSDME, and ACTB in **(E)** U87MG and U251 pretreated with 3-MA (10 mM), followed by exposure to 150 μM GG or DMSO for another 48 h. All data are expressed as the mean ± SD of values from experiments performed in triplicate. ***P < 0.001 compared to controls.

The mammalian target of rapamycin (mTOR) is a protein serine/threonine kinase and a key regulator of autophagy. (mTOR senses the levels of intracellular ATP, growth factors, and insulin, and thus, changes in intracellular nutrition and energy (28). Thus, we examined whether mTOR and other proteins in the pathway were involved in GG-induced autophagy in GBM cells. We first examined mTOR, which became dephosphorylated at Ser2448 in GG-treated U87MG cells and indicated that induction of autophagy by GG was mTOR-dependent (Figure 5A and Supplementary Figure 5A). Previous studies have shown that GG activates AMP-Activated protein kinase (AMPK) (11), which suppresses mTOR and thus enhances autophagy flux. AMPK is a heterotrimeric complex composed of an α catalytic subunit, a β regulatory subunit and a γ regulatory subunit with phosphorylation of the AMPKα at the Thr172 site which is essential for AMPK activation. Phosphorylated AMPKα Thr172 was significantly increased in GG-treated U87MG cells (Figure 5A). To further verify that GG-induced autophagy was AMPK dependent, U87MG cells were treated with GG and the AMPK inhibitor Compound C (20 μM) (Selleck Chemicals, TX, USA) for 48 h. Compound C treatment led to reduced levels of P-AMPKα Thr172 and attenuated GG-induced autophagy flux, as determined by decreased levels of MAP1LC3B-II (Figure 5B and Supplementary Figure 5B). We also detected downstream molecules of AMPKα as energy receptors, such as ACC, Phosphorylated ACC (Ser79), PDK2 (Thr172), HMGCR. Their changes also confirmed the activation of the AMPK pathway (Figures 5C,D and Supplementary Figure 5C). These results indicated that the AMPK/mTOR pathway was involved in GG-induced autophagy in GBM cells.

### Inhibition of Autophagy Enhances Aggravates GG-Induced Apoptosis and Pyroptosis in GBM Cells

Current research has demonstrated that the relationship between autophagy and apoptosis can be mutually exclusive or coordinated in programmed cell death (29, 30). We therefore investigated the relationship between autophagy, apoptosis, and pyroptosis in GBM cells under treatment with 150 μM GG. On western blotting analysis, apoptosis-related proteins, such as Bax and cleaved-PARP1, as well as the pyroptosis-related protein N-GSDME, were increased in GG-treated U87MG and U251 cells in the presence of 3-MA, an inhibitor of autophagy (Figure 5E and Supplementary Figure 5D). Taken together, 150 μM GG simultaneously induced apoptosis, pyroptosis, and protective autophagy in GBM cells in culture.

### GG Inhibits Growth of GBM Cells *in vivo*

**Figure 6:**
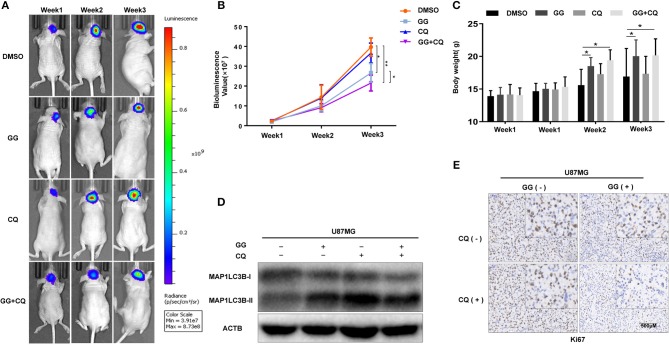
GG inhibits tumor growth in an orthotopic model for GBM in mice. **(A)** U87MG cells expressing luciferase were orthotopically implanted into athymic nude mice, and tumor growth was monitored using the PerkinElmer IVIS Spectrum for detection of bioluminescence. Bioluminescent signals were measured at days 7, 14, and 21 after implantation. **(B)** Bioluminescence values plotted as a function of time in days to assess tumor growth (days 7, 14, and 21). **(C)** Graphic representation of results from weight of athymic mice in each experimental group (days 0, 7, 14, and 21). **(D)** Western blotting analysis performed on lysates (20 μg) prepared from xenografts to detect protein levels of MAP1LC3B and ACTB in each experimental group. **(E)** Images of immunohistochemical staining for Ki67 in tumors from each group as indicated (scale bars: 50 μm). All data are expressed as the mean ± SD of values from experiments performed in triplicate. *P < 0.05 and **P < 0.01 compared between the 2 treatments.

The therapeutic efficacy of GG was assessed in an orthotopic tumor model derived from U87MG-luciferase expressing cells implanted in athymic mice. Tumor growth was evaluated using luciferase bioluminescence. GG treatment significantly suppressed tumor growth relative to vehicle control in tumor bearing mice (at 3 weeks, ~25 × 10^7^ vs. ~ 40 × 10^7^ photons/s, GG vs. vehicle control; Figures 6A,B and Supplementary Figure 6). The combined administration of GG and CQ was more effective compared to GG monotherapy (~20 × 10^7^ vs. ~25 × 10^7^ photons/s, GG+CQ vs. GG; Figures 6A,B). However, there was no significant difference between CQ treated animals and controls. The weight of GG and GG + CQ-treated animals were also increased relative to controls at 2 and 3 weeks following initiation of treatment (*P* < 0.05; Figure 6C). Tissue protein immunoblotting yielded similar results to those in GBM cell lines (Figure 6D).

Immunohistochemistry performed on tissue sections from xenografts also demonstrated that Ki67, a marker of cell proliferation, was decreased in GG and GG + CQ-treated tumors compared to untreated controls (Figure 6E). Therefore, GG suppressed tumor growth *in vivo*, and combined treatment with an inhibitor of autophagy enhanced GG-induced tumor growth inhibition.

## Discussion

The antineoplastic effect of GG has been observed in a variety of tumors, including leukemia (31), colon cancer (32), retinoblastoma (33), and breast cancer (34). As a natural medicinal extract, GG exhibits low toxicity to the animal and non-specificity, with regard to tumor tissues, which differs from chemically synthesized drugs. Therefore, a molecular understanding of the antineoplastic characteristics of GG might be of value in the treatment of human GBM, which responds poorly to current therapeutic approaches. GG has been shown to inhibit cell migration and invasion of the GBM cell line A172 under non-toxic doses depending on its ability to activate ADAM9 and Erk1/2 (35). Nonetheless, our experiments paid attention to the phenomenon that GG induces not only apoptosis and pyroptosis, which inhibit GBM growth *in vitro* and *in vivo*, but also autophagy.

**Figure 7:**
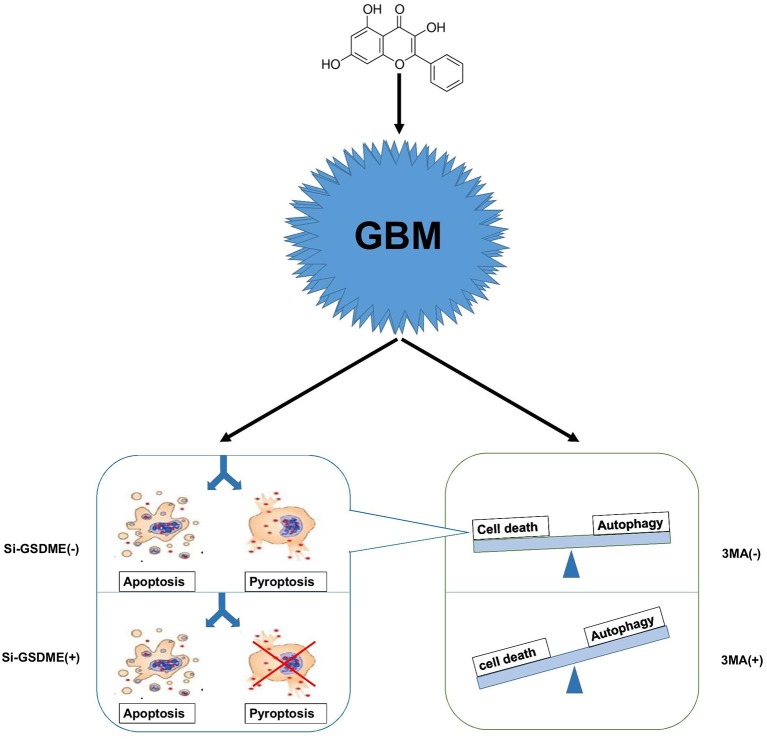
A hypothetical mechanism of GG exerted antitumor activity in GBM cells. GG induces autophagy through the AMPK/mTOR signaling pathway and induces mitochondrial apoptosis and pyroptosis in GBM cells. When autophagy is blocked, apoptosis, and pyrosis increase significantly. Crosstalk between apoptosis and pyrosis may exist due to mediation of the processes by some of the same proteins.

Autophagy is a cellular process that is extremely conserved in evolution. When cells are under stress due to energy levels incompatible with growth/survival, autophagy is the process whereby organelles and proteins are digested into amino acids and essentially recycled to maintain cell survival. Continuous cellular proliferation, insufficient blood supply, aerobic glycolysis and infiltration of inflammatory cells render tumor cells relatively energy-deficient so that they maintain a high level of autophagy. Lack of energy is often accompanied by hypoxia, which is a hallmark of GBM. Hypoxia induces autophagy as a mechanism of protection and survival. Therefore, tumor cells tend to engage autophagy for various reasons. Many antitumor treatments, including chemotherapy, radiation therapy, and common drugs, have been reported to modulate cellular autophagy (36, 37). In the case of GG, the treatment may induce energy stress in GBM cells and thus, autophagy; tumor cells face the choice between survival and death. In our work, we prefer to consider this state as the damage state. Survival requires maintaining normal organelle function and a sufficient energy supply. Cell death is a process that also requires energy and generation of the necessary components. The energy produced through autophagy may therefore be used to prepare for either cell survival or cell death. Therefore, to consider autophagy as only a protective function might not be sufficient, and we cannot simply assume that drugs inducing autophagy are always beneficial to tumor cells. So-called protective autophagy might simply halt the damage state, and thus protect cells from proceeding down a cell death pathway (Figure 7).

While crosstalk between apoptosis and autophagy is well-established, the relationship between autophagy and pyroptosis remains poorly defined. Although it was initially identified in bacterial immunity, pyroptosis has become an increasingly acknowledged form of programmed cell death occurring in biological scenarios including tumor therapy and chronic inflammation. Many studies have shown that GSDME is highly expressed in normal tissues but silenced in cancers due to promoter hypermethylation. This pattern of expression is consistent with a role as a putative tumor suppressor (38, 39). In GBM, however, the situation is reversed; GSDME is highly expressed relative to normal tissue. Increased expression of GSDME may represent a unique opportunity to exploit pyroptosis in the treatment of GBM. When we blocked autophagy *in vitro*, pyroptosis increased in GBM cells. *In vivo*, combination therapy in xenograft models in mice significantly improved survival. Our studies confirm that antineoplastic drugs combined with autophagy inhibitors provide a basis for cocktail therapy. Pyroptosis differs from apoptosis, however, in a critical aspect; the cell membrane is damaged by the N-terminal region of GSDME during pyroptosis, which releases cellular contents into the extracellular environment. The released cellular contents have the potential to stimulate inflammation and may initiate an anti-tumor immune response. Thus, pyroptosis may have synergistic effects with current anti-tumor immunotherapy. In addition, certain tumor cells have anti-apoptosis mechanisms, and the existence of pyroptosis pathway may be an important way for drugs to kill tumor cells.

In summary, we have examined the role of apoptosis and pyroptosis, two mechanisms which induce programmed cell death, in GG-induced inhibition of GBM cell growth, and found that the molecule simultaneously activates both processes. However, data from other studies is controversial. Some studies have demonstrated that pyroptosis suppresses the apoptotic pathway in macrophages (40, 41), while in some cell lines, the process has been shown to occur as secondary necrosis after apoptosis (24). Recent reports have however confirmed that GSDME is a critical substrate of caspase-3 and a key mediator of non-immune cell pyroptosis (24). We thus propose that GG treatment induces concomitant apoptosis and pyroptosis at the molecular level through the same upstream pathway, activation of caspase-3, and therefore, the two processes may interact for efficient execution of cell death in response to treatment. Future studies are thus warranted to determine the relative contribution of these two processes to the anti-neoplastic effects of GG as well as the specific molecules involved.

## Data Availability Statement

All data included in this study are available upon request by contact with the corresponding author.

## Ethics Statement

All animal procedures were approved by the Institutional Animal Care and Use Committee (IACUC) of Shandong University (Jinan, China).

## Author Contributions

JW, XL,YK, ZF, QQ, and JwW contributed to the conception of the study. SW, MH, YZ, and XZ contributed to experimental technology and experimental design. QZ provided molecular biology experimental technical support. BH, NY, WL, GX, and DZ performed the data analyses and animal experiment guide. AC helped performthe analysis with constructive discussions and paper modification. YK, JW, and XL wrote the manuscript.

### Conflict of Interest

The authors declare that the research was conducted in the absence of any commercial or financial relationships that could be construed as a potential conflict of interest.

## Supplementary Material

Supplementary Figure 1 **(A)** Molecular structure of GG. **(B)** Image of colony formation assays in U87MG and U251 after treatment with the indicated concentrations of GG for 2 weeks. Cells were fixed and stained with crystal violet. **(C)** Graphic representation of cell cycle distribution (PI) analyzed by flow cytometry for U251 and U87MG cells treated with 150 μM GG or DMSO for 48 h.

Supplementary Figure 2 **(A)** Western blotting analysis of lysates (20 μg) prepared from U87MG and U251 cells treated with DMSO or GG at the concentrations indicated for 48 h. Membranes were incubated with antibodies against CDH2, MMP2, CDK4, CCND1, P21, PCNA, and ACTB (protein loading control). **(B)** Quantitation of protein levels of CDH2, MMP2, CDK4, CCND1, P21, PCNA, and ACTB. **(C)** Quantitation of protein levels of cleaved-PARP1, Bcl-2, BAX, and ACTB in Figure 2C. ^*^*P* < 0.05 and ^**^*P* < 0.01 compared to controls.

Supplementary Figure 3 **(A)** Graphic representation of the mRNA expression of *GSDME* in glioma and Non-tumor in the Rembrandt database. **(B)** Kaplan–Meier survival curves for glioma patients with higher expression of GSDME and lower expression of GSDME. **(C)** Quantitation of protein levels of N-GSDME and ACTB of Figure 3C. **(D)** Graphic representation of LDH Release Assay in U87MG and U251 cells with knock-down of GSDME compared to controls. **(E)** Quantitation of protein levels of GSDME and ACTB of Figure 3F. **(F)** Graphic representation of Cell Counting Kit-8 between control and knockdown of GSDME in U87MG and U251. Quantitative histogram of C-PARP-1 and P-H2A.X (Ser139) in U87MG **(G)** and U251 **(H)** cells. ^*^*P* < 0.05, ^**^*P* < 0.01, ^***^*P* < 0.001, and ^****^*P* < 0.0001 compared to controls.

Supplementary Figure 4 **(A)** Western blotting analysis performed to detect levels of MAP1LC3B and ACTB in U251 treated with 3-MA (5 mM) for 20 min, followed by exposure to 150 μM GG or DMSO for another 48 h. **(B)** Western blotting analysis performed to detect levels of MAP1LC3B and ACTB in U251 CQ (10 μM) for 20 min, followed by exposure to 150 μM GG or DMSO for another 48 h. **(C)** Quantitative histogram of Figure 4C. Quantitation of protein levels of MAP1LC3B-II, SQSTM1 and ACTB in U87MG **(D,E)** and U251 **(F,G)** cells after corresponding treatment. ^*^*P* < 0.05, ^**^*P* < 0.01, ^***^*P* < 0.001, and ^****^*P* < 0.0001 compared to controls.

Supplementary Figure 5 **(A)** Quantitative of protein levels of AMPK-a, P-AMPK-a (Thr172), mTOR, P-mTOR (Ser2448) and MAP1LC3B-II and ACTB in U87MG and U251 after exposure to 150 μM GG or DMSO for 48 h. **(B)** Quantitative histogram of Figure 5B. **(C)** Quantitative of protein levels of AMPK-a, P-AMPK-a (Thr172), P-ACC (Ser79), PDK2, HMGCR treated with DMSO or GG (150 μM) in in U87MG and U251. **(D)** Quantitative histogram of Figure 5E. ^*^*P* < 0.05, ^**^*P* < 0.01, ^***^*P* < 0.001, and ^****^*P* < 0.0001 compared to controls.

Supplementary Figure 6 Tumor growth was monitored using the PerkinElmer IVIS Spectrum for detection of bioluminescence. Bioluminescent signals were measured at days 7, 14, and 21 after implantation.
