# Supplementary figures and images for: The Natural Flavonoid Galangin Elicits Apoptosis, Pyroptosis, and Autophagy in Glioblastoma
Source: Front Oncol. 2019 Sep 27;9:942. doi: 10.3389/fonc.2019.00942 (PMC6776614; doi:10.3389/fonc.2019.00942)

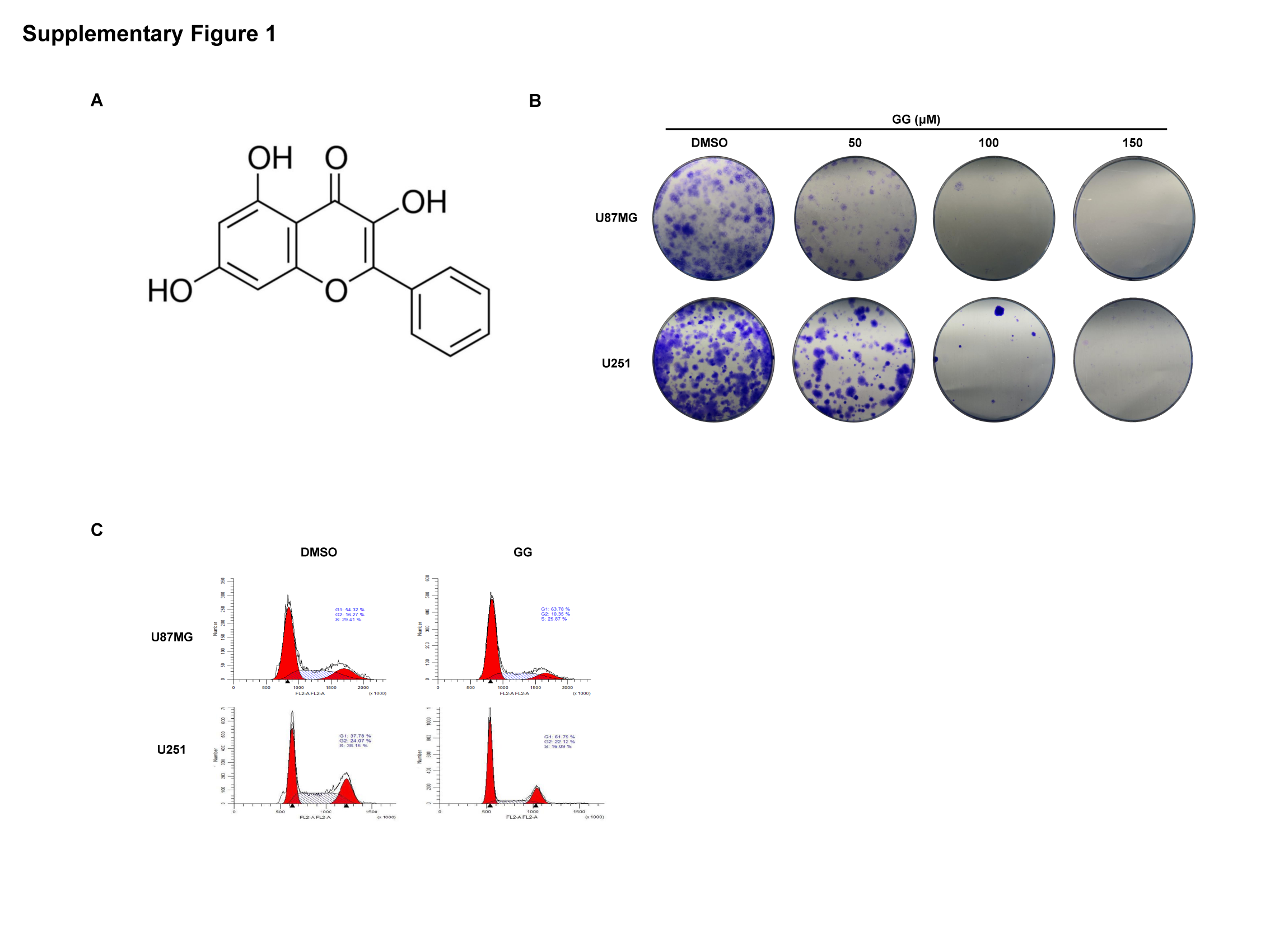

Supplement: Supplementary Figure 1 — (A) Molecular structure of GG. (B) Image of colony formation assays in U87MG and U251 after treatment with the indicated concentrations of GG for 2 weeks. Cells were fixed and stained with crystal violet. (C) Graphic representation of cell cycle distribution (PI) analyzed by flow cytometry for U251 and U87MG cells treated with 150 μM GG or DMSO for 48 h. [file Image_1.TIF]

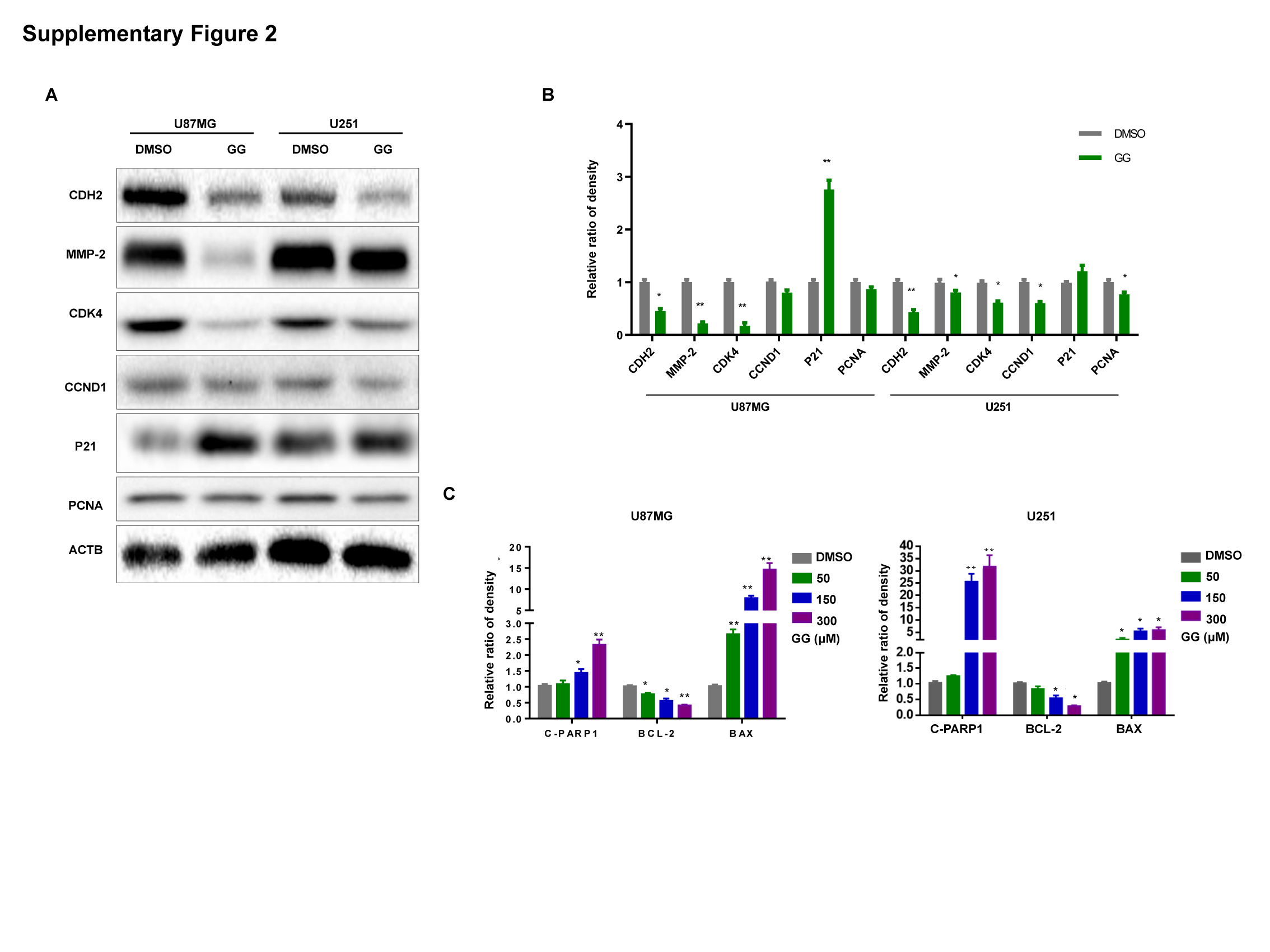

Supplement: Supplementary Figure 2 — (A) Western blotting analysis of lysates (20 μg) prepared from U87MG and U251 cells treated with DMSO or GG at the concentrations indicated for 48 h. Membranes were incubated with antibodies against CDH2, MMP2, CDK4, CCND1, P21, PCNA, and ACTB (protein loading control). (B) Quantitation of protein levels of CDH2, MMP2, CDK4, CCND1, P21, PCNA, and ACTB. (C) Quantitation of protein levels of cleaved-PARP1, Bcl-2, BAX, and ACTB in Figure 2C. *P < 0.05 and **P < 0.01 compared to controls. [file Image_2.TIF]

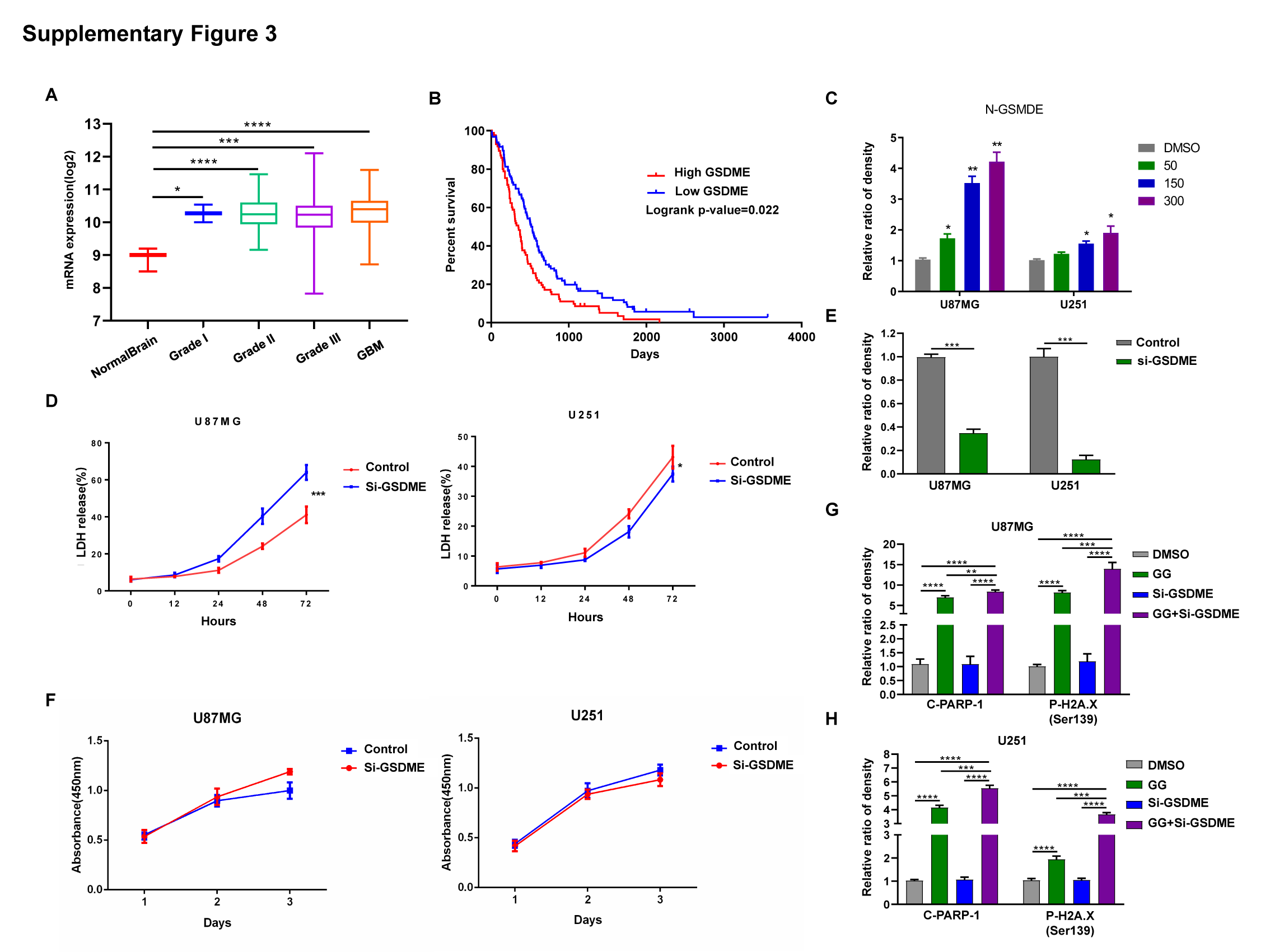

Supplement: Supplementary Figure 3 — (A) Graphic representation of the mRNA expression of GSDME in glioma and Non-tumor in the Rembrandt database. (B) Kaplan–Meier survival curves for glioma patients with higher expression of GSDME and lower expression of GSDME. (C) Quantitation of protein levels of N-GSDME and ACTB of Figure 3C. (D) Graphic representation of LDH Release Assay in U87MG and U251 cells with knock-down of GSDME compared to controls. (E) Quantitation of protein levels of GSDME and ACTB of Figure 3F. (F) Graphic representation of Cell Counting Kit-8 between control and knockdown of GSDME in U87MG and U251. Quantitative histogram of C-PARP-1 and P-H2A.X (Ser139) in U87MG (G) and U251 (H) cells. *P < 0.05, **P < 0.01, ***P < 0.001, and ****P < 0.0001 compared to controls. [file Image_3.TIF]

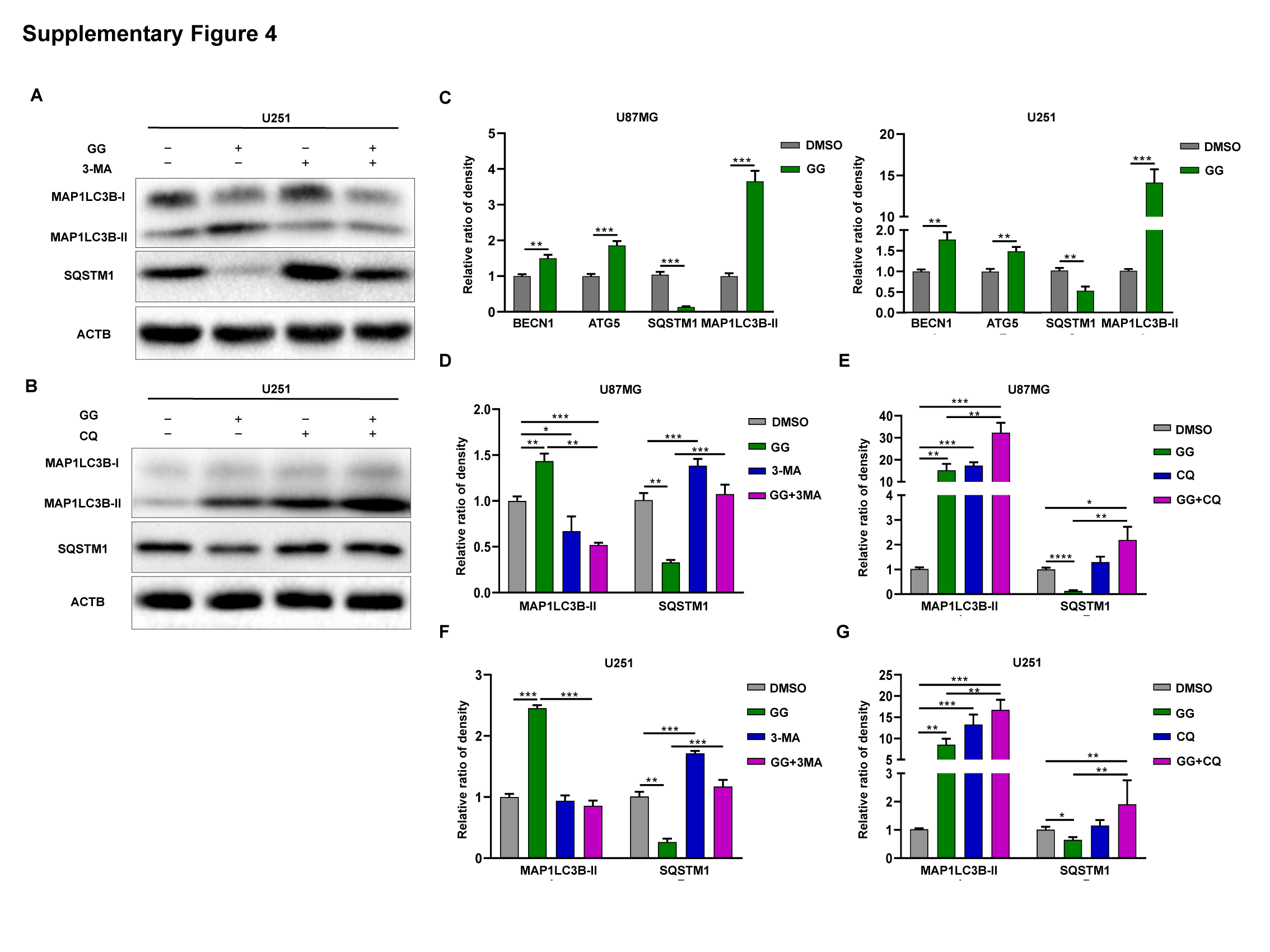

Supplement: Supplementary Figure 4 — (A) Western blotting analysis performed to detect levels of MAP1LC3B and ACTB in U251 treated with 3-MA (5 mM) for 20 min, followed by exposure to 150 μM GG or DMSO for another 48 h. (B) Western blotting analysis performed to detect levels of MAP1LC3B and ACTB in U251 CQ (10 μM) for 20 min, followed by exposure to 150 μM GG or DMSO for another 48 h. (C) Quantitative histogram of Figure 4C. Quantitation of protein levels of MAP1LC3B-II, SQSTM1 and ACTB in U87MG (D,E) and U251 (F,G) cells after corresponding treatment. *P < 0.05, **P < 0.01, ***P < 0.001, and ****P < 0.0001 compared to controls. [file Image_4.TIF]

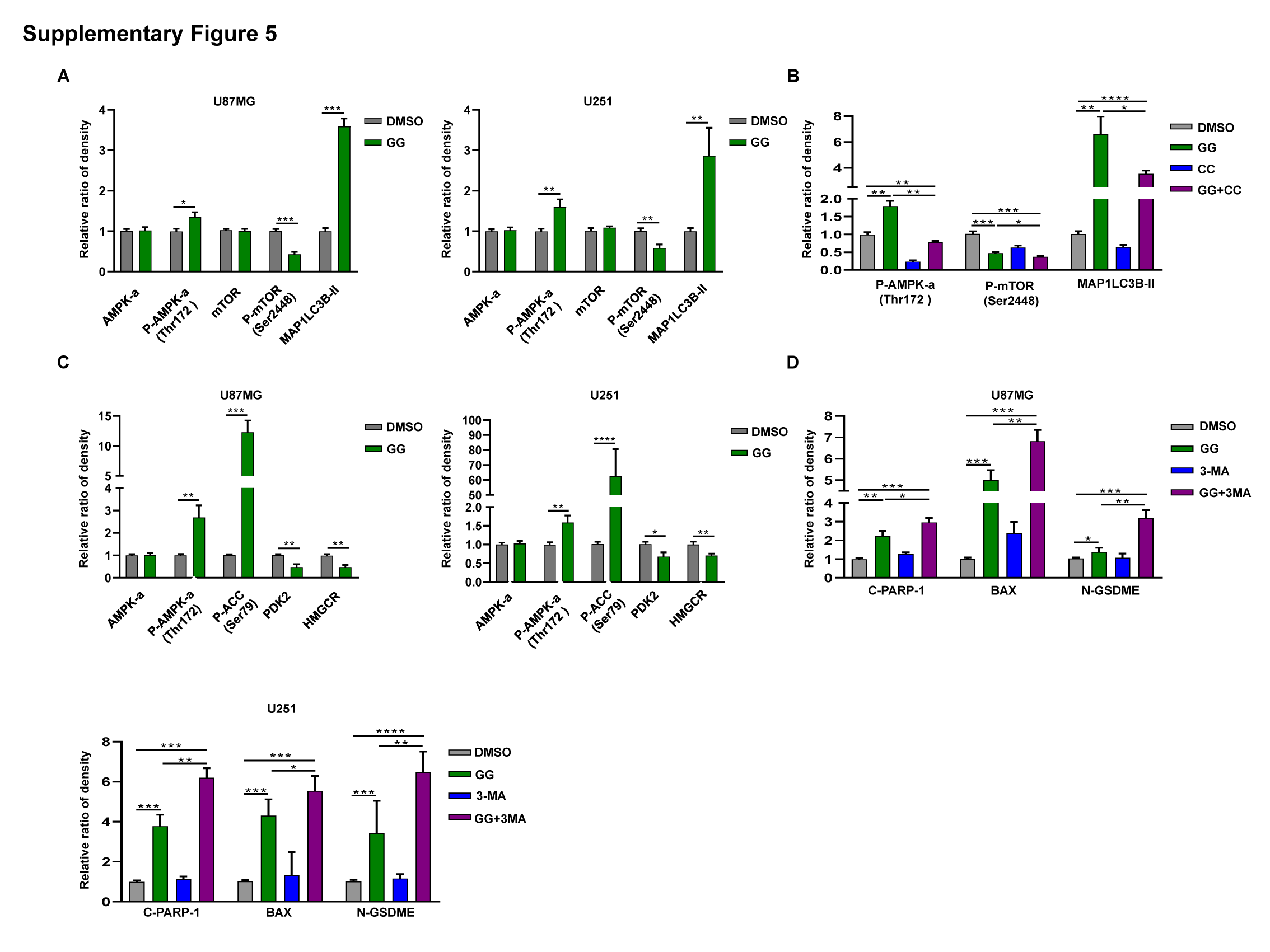

Supplement: Supplementary Figure 5 — (A) Quantitative of protein levels of AMPK-a, P-AMPK-a (Thr172), mTOR, P-mTOR (Ser2448) and MAP1LC3B-II and ACTB in U87MG and U251 after exposure to 150 μM GG or DMSO for 48 h. (B) Quantitative histogram of Figure 5B. (C) Quantitative of protein levels of AMPK-a, P-AMPK-a (Thr172), P-ACC (Ser79), PDK2, HMGCR treated with DMSO or GG (150 μM) in in U87MG and U251. (D) Quantitative histogram of Figure 5E. *P < 0.05, **P < 0.01, ***P < 0.001, and ****P < 0.0001 compared to controls. [file Image_5.TIF]

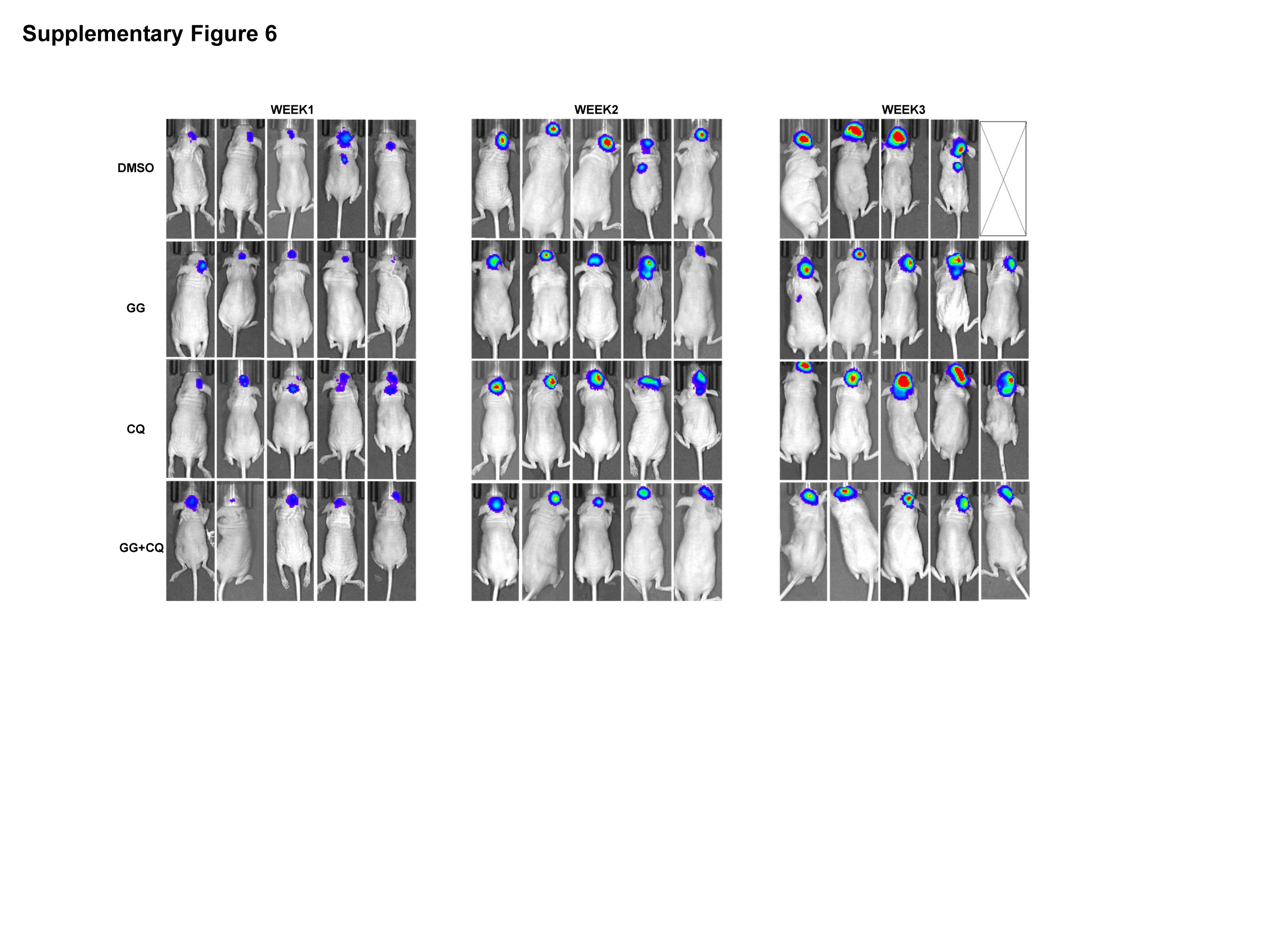

Supplement: Supplementary Figure 6 — Tumor growth was monitored using the PerkinElmer IVIS Spectrum for detection of bioluminescence. Bioluminescent signals were measured at days 7, 14, and 21 after implantation. [file Image_6.TIF]
